# Geometric changes of parotid glands caused by hydration during chemoradiotherapy

**DOI:** 10.1186/s13014-015-0554-x

**Published:** 2015-12-01

**Authors:** Petronella M. Kager, Sanne C. C. van Weerdenburg, Simon R. van Kranen, Suzanne van Beek, Elisabeth A. Lamers-Kuijper, Wilma D. Heemsbergen, Olga Hamming-Vrieze, Peter Remeijer

**Affiliations:** Department of Radiation Oncology, The Netherlands Cancer Institute, Plesmanlaan 121, 1066 CX Amsterdam, The Netherlands; Division of Medical Technology, Inholland University of Applied Sciences, Bijdorplaan 15, 2015 CE Haarlem, The Netherlands

**Keywords:** Head and neck cancer, Parotid glands, Anatomy changes, Adaptive radiotherapy

## Abstract

**Background:**

Plan adaptation during the course of (chemo)radiotherapy of H&N cancer requires repeat CT scanning to capture anatomy changes such as parotid gland shrinkage. Hydration, applied to prevent nephrotoxicity from cisplatin, could temporarily alter the hydrogen balance and hence the captured anatomy. The aim of this study was to determine geometric changes of parotid glands as function of hydration during chemoradiotherapy compared to a control group treated with radiotherapy only.

**Methods:**

This study included an experimental group (*n* = 19) receiving chemoradiotherapy, and a control group (*n* = 19) receiving radiotherapy only. Chemoradiotherapy patients received cisplatin with 9 l of saline solution during hydration in the first, fourth and seventh week. The delineations of the parotid glands on the planning CT scan were automatically propagated to Cone Beam CT scans using deformable image registration. Relative volume and position of the parotid glands were determined at the second chemotherapy cycle (week four) and at fraction 35.

**Results:**

When saline solution was administrated, the volume temporarily increased on the first day (7.2 %, *p* < 0.001), second day (10.8 %, *p* < 0.001) and third day (7.0 %, *p* = 0.016). The gland positions shifted lateral, the distance between glands increased on the first day with 1.5 mm (*p* < 0.001), on the second day 2.2 mm (*p* < 0.001). At fraction 35, with both groups the mean shrinkage was 24 % ± 11 % (1SD) and the mean medial distance between the parotid glands decreased by 0.47 cm ± 0.27 cm.

**Conclusions:**

Hydration significantly modulates parotid gland geometry. Unless, in the context of adaptive RT, a repeat CT scan is timed during a chemotherapy cycle, these effects are of minor clinical relevance.

## Background

Over the last few decades, chemoradiotherapy (CRT) became the standard of care in patients with locally-advanced head and neck (H&N) cancer (stage III/IV) aiming at organ and function preservation. With CRT, a cytostatic is used as a radiosensitizer enhancing the damaging effect of radiotherapy (RT).

Cisplatin is a radiosensitizer that is often used in CRT of H&N cancer. Besides its radiosensitizing effect, cisplatin is known to have many side effects. Nephrotoxicity is one important example. To reduce the risk of nephrotoxicity, a substantial amount of saline solution is administered to the patient before and after the administration of cisplatin. This hydration during CRT of H&N cancer can potentially alter the anatomy of the patient.

Several authors [1–9] described geometrical changes of organs-at-risk (OARs) during treatment. The organ most studied is the parotid gland (PG), showing a decrease of volume during treatment with a medial shift of the center-of-mass [1–8]. The study of Bhide et al. [1] demonstrated that the largest volume decrease occurs in the second week of treatment. No significant difference was distinguished between the volume decrease of the ipsi- and contralateral PG. Several studies pointed out that the medio-lateral distance between the parotid glands (PGs) decreases [1, 2, 6–8]. This shift sets in during the second week of treatment with the largest displacement during the fourth week [1].

Preliminary investigations on PG geometrical variability in our institute showed an occasional increase of the PG volume on Cone Beam CT (CBCT) scans of H&N cancer patients treated with CRT. These patients were all treated with concomitant cisplatin and received hydration. Based on these findings it was hypothesized that the geometry of the PGs is influenced by hydration during CRT and this comparative study was designed.

Generally, the mean dose to the PGs is kept below 26Gy to prevent long term xerostomia and preserve quality of life. The volume decrease and medial shift of the PGs as described above, result in an increase of the delivered dose [1, 3–6, 10]. Recently, adaptive RT has emerged [11–13] where a repeat CT scan is acquired and the treatment plan is adjusted to account for these or other relevant geometrical changes and reduce toxicity. However, if the geometry of the PGs is influenced by hydration, this needs to be taken into account, in deciding when to make a repeat CT scan for adaptive RT.

The aim of this study was to determine geometric changes of PGs as a function of hydration during CRT compared to a control group treated with RT only.

## Methods

### Patient and treatment characteristics

This retrospective study included 38 patients treated for H&N cancer: an experimental group (*n* = 19) receiving CRT and a control group (*n* = 19) receiving RT only. Patients with daily CBCT scans and the standard chemotherapy regime were selected. Patients in the CRT group received cisplatin with hydration in the first, fourth and seventh week of the treatment according to the RADPLAT intravenous protocol: with each chemotherapy cycle, 100 mg/m^2^ cisplatin with about 9 l of saline solution during hydration was administrated intravenously in three days.

Both groups received a total dose of 70Gy on tumor site in 35 fractions of 2Gy. For the CRT group, these fractions were given in 7 weeks. Most patients in the RT alone group received RT with an accelerated scheme of 6 weeks: once a week 2 fractions a day separated by a time interval of at least 6 h. Further patient and treatment characteristics are described in Table 1.

**Table 1:** Patient and treatment characteristics

Patient characteristics		CRT (*n* = 19)	RT (*n* = 19)	*p*
Gender	Male	14	11	0.495
	Female	5	8	
Age in years	Mean (range)	58 (42–71)	62 (47–81)	0.254
Tumor site	Nasopharynx (T3-T4)	2	1	0.324
	Oropharynx (T1-T4)	15	13	
	Hypopharynx (T2-T4)	2	2	
	Larynx (T2-T3)	0	3	
pCT: PG volume (cm^3^)	Mean (range)	20 (7–47)	24 (13–44)	0.082
pCT: medio-lateral distance between PGs (cm)	Mean (range)	12 (10–13)	11 (10–13)	0.358
Planned dose ipsilateral PG (Gy)	Mean	40.7	34.5	0.148
	SD	11.7	12.2	
Planned dose contralateral PG (Gy)	Mean	27.2	23.0	0.122
	SD	8.1	7.9	
Body weight	Mean pre-treatment (kg)	70	78	0.373
	Mean loss (%)	7.9	5.6	
Tube feeding	Number of patients	15	6	0.008

*CRT* chemoradiotherapy group, *RT* radiotherapy alone group, *pCT* planning CT, *PG(s)* parotid gland(s)

### Treatment delivery and Cone Beam CT guidance protocol

All patients were positioned using a thermoplastic mask (Orfit Industries, Wijnegem, Belgium), a standard headrest and a knee support (Civco Medical Solutions, Kolona, USA). The planning CT (pCT) scan (Hispeed LX/I, General Electric Medical Systems, Milwaukee, WI, USA or Somatom Sensation Open, Siemens AG, Erlangen, Germany) was acquired with a slice distance and thickness of 3 mm from the cranium to the sternum. The patient setup on the linear accelerator (Elekta, Synergy, Crawley, UK) was verified by registration of the bony anatomy from the pCT to the CBCT using chamfer matching and was corrected using a regular offline protocol [14]. Additional CBCT scans were acquired for research purposes within the context of other studies. A total of 1170 CBCT scans were evaluated for this study with an average of 31 CBCT scans per patient. CBCT scans were acquired over a 360° arc in 1 min (16 mA – 40 ms – 120 kV, per projection) with an isocenter dose of ~1 cGy.

### Volume and center-of-mass

For each patient, the PGs were delineated on the pCT scan. PG geometry during treatment was determined in the CBCT scans by propagating these delineations using deformable image registration (DIR). First the CBCT scan was rigidly registered with the pCT scan on local bony structures near the PGs. Next, soft tissue deformations were determined with DIR. An in-house developed BSpline deformable registration algorithm was used [15]. The accuracy and precision of this algorithm is reviewed in the discussion. Finally, the deformation fields were used to propagate the delineations of the PGs from the pCT to the CBCT scan.

Volume and center-of-mass (COM) of both PGs were determined with the propagated delineations of the PGs on the CBCT scans. The COM was given by x-, y- and z-coordinates corresponding to the medio-lateral, dorso-ventral and cranio-caudal direction.

### Data analysis

Volume and position parameters of the PGs were calculated. Volume change was evaluated by dividing the PG volume on the CBCT scan by the volume on the pCT scan. According to Bhide et al. [1] no difference between the volume decrease of the ipsi- and contralateral PG is expected. Also both PGs received the same amount of hydration. Therefore, we hypothesized that volumes could be combined and the average volume of the two PGs was calculated.

To obtain information on positional changes, the 3D distance between the left and right PG was calculated by subtracting the COMs. Subsequently, the distance between the PGs on the pCT scan was subtracted from the distance on the CBCT scan. In this manner the pCT was used as a reference to calculate relative volume and position of the PGs with all CBCT scans that were acquired during treatment.

### Statistical analysis

The Mann–Whitney *U*-test was used to test for statistical difference between relative volume of the ipsi- and contralateral PG at treatment day 19 (just prior to the second chemotherapy cycle). Baseline patient and treatment characteristics (Table 1) were tested for statistical difference between the CRT and RT alone group with the Fisher’s Exact, Pearson Chi-Square or Mann–Whitney *U*-test.

Relative volume and position of the PGs were tested for statistical differences between the CRT and RT alone group around the second chemotherapy cycle (week four) and at the 35th fraction. For a statistical analysis there was not enough information with the first chemotherapy cycle (week 1: no CBCT scans prior to hydration) and the third chemotherapy cycle (week 7: not sufficient CBCT scans in the RT alone group due to the accelerated 6 week scheme for most of these patient). For the analysis around the second chemotherapy cycle, the starts of hydration for all CRT patients were redefined as day 0 (usually Sundays). For each patient, the difference in relative volume and position between the days after start of hydration (day 1 till day 5) and the reference day (day −3; three days before the start of hydration; baseline volume) was calculated. The CRT group and RT alone group were tested for statistical difference with a Mann–Whitney *U*-test. *P*-values <0.05 were considered significant.

## Results

**Fig. 1:**
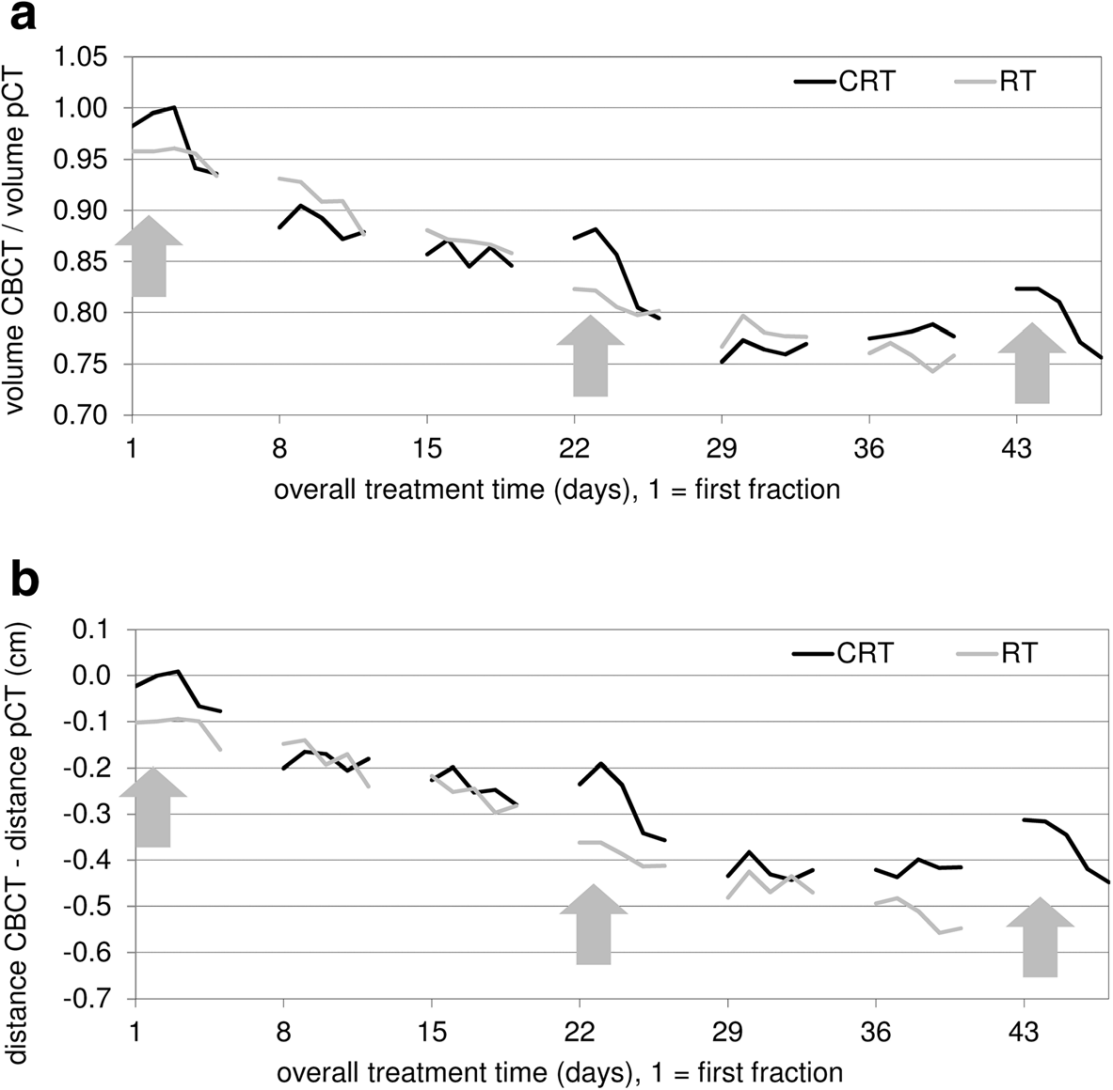
Overall volume and positional change of the parotid glands. Volumetric change of the parotid glands (PGs) (**a**) and distance change between the PGs in medio-lateral direction (**b**) from the start of treatment. The black line corresponds to the average of the chemoradiotherapy (CRT) group, the grey line corresponds to the average of the radiotherapy alone (RT) group. RT starts at treatment day 1. Saline solution was administered during the first, second and third chemotherapy cycle, respectively during the first, fourth and seventh week of treatment (*arrows*). Most patients in the RT alone group received RT with an accelerated scheme, therefore the grey line stops at week 6

Relative volume and position of the PGs during treatment are presented in Fig. 1. In general, most patients received their first fraction (treatment day 1) on Monday, consequently there is too little data available on treatment day 6, 7, 13, 14, etc. representing weekend days. Some patients received their first fraction (treatment day 1) on Tuesday or Wednesday, consequently the number of patients per treatment day with available CBCT scan varies to at least 10.

At treatment day 19, just prior to the second chemotherapy cycle, the mean ipsilateral PG volume loss was 16 % ± 11 % (1SD) and the mean contralateral PG volume loss was 14 % ± 9 %. There was no significant difference between the relative volume of the ipsi- and contralateral PG just prior to the second chemotherapy cycle.

Table 1 presents patient and treatment characteristics and summarizes the outcome of the statistical analysis. The number of patients in the CRT group receiving tube feeding was significantly higher compared to the RT alone group (*p* = 0.008). Concerning other patient and treatment characteristics there were no significant differences between the CRT and RT alone group.

**Fig. 2:**
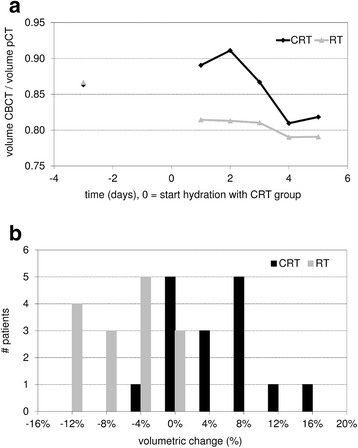
Volume change of the parotid glands during the fourth week of treatment. Mean parotid gland volume for the chemoradiotherapy (CRT) group and the radiotherapy alone (RT) group, relative to the start of hydration (renamed day 0) during the second chemotherapy cycle of the CRT group in the fourth week of treatment (**a**). Volumetric change of the parotid glands between 3 days prior and 2 days after starting hydration within the CRT group and the RT group (**b**)

**Fig. 3:**
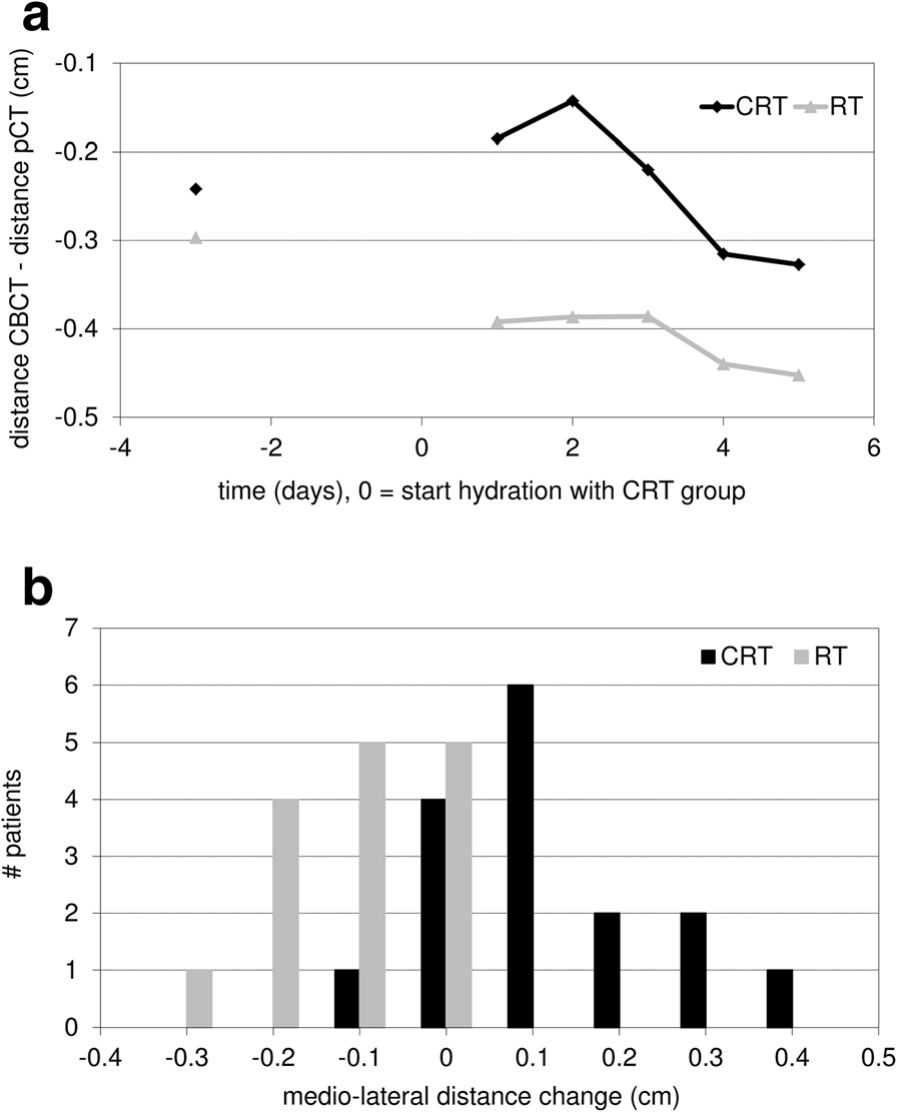
Positional change of the parotid glands during the fourth week of treatment. Mean distance change between the parotid glands (PGs) in medio-lateral direction for the chemoradiotherapy (CRT) group and the radiotherapy alone (RT) group, relative to the start of hydration (renamed day 0) during the second chemotherapy cycle of the CRT group in the fourth week of treatment (**a**). Distance change between the PGs in medio-lateral direction between 3 days prior and 2 days after starting hydration within the CRT group and the RT group (**b**)

At the intervals where saline solution was administered, the volume of the PGs within the CRT group increased temporarily and the position showed a temporal shift to a more lateral position. Table 2 presents results of both patient groups at the time interval around the second chemotherapy cycle (week four). Mean and median values are comparable, indicating probably normally distributed data. Table 2 also summarizes the outcome of the statistical analysis. The relative change in volume of the PGs was significantly different between groups on the first day (7.2 %, *p* < 0.001), the second day (10.8 %, *p* < 0.001) and the third day (7.0 %, *p* = 0.016) after starting hydration (day 0) (Fig. 2). Maximum increase of the mean PG volume was seen on the second day after starting hydration with 5.1 %. The relative change in position of the PGs in medio-lateral direction was significantly different between groups on the first (1.5 mm, *p* < 0.001) and second day (2.2 mm, *p* < 0.001) after starting hydration (Fig. 3). Due to hydration the movement in medial direction was reversed temporarily. No obvious shifts in dorso-ventral and cranio-caudal directions were noticed.

**Table 2:** Effect of hydration on parotid gland volume and position during chemoradiotherapy

	PG volume change relative to day_−3_ (%)						
	CRT		RT		CRT – RT		
	Mean	Median	Mean	Median	Mean	Median	*p*
day_−3_	86.3	87.7	86.7	87.1			
day_1_ – day_−3_	+2.1	+1.8	−5.1	−4.5	7.2	6.3	<0.001
day_2_ – day_−3_	+5.1	+5.4	−5.6	−3.8	10.8	9.2	<0.001
day_3_ – day_−3_	+1.4	−1.8	−5.6	−4.7	7.0	2.8	0.016
day_4_ – day_−3_	−5.4	−6.9	−7.7	−7.3	2.2	0.4	0.601
day_5_ – day_−3_	−4.8	−4.8	−6.8	−6.7	2.0	1.9	0.511
	Medio-lateral distance change between PGs relative to day_−3_ (cm)						
	CRT		RT		CRT – RT		
	Mean	Median	Mean	Median	Mean	Median	*p*
day_−3_	−0.24	−0.25	−0.30	−0.31			
day_1_ – day_−3_	+0.06	+0.04	−0.09	−0.09	0.15	0.13	<0.001
day_2_ – day_−3_	+0.12	+0.09	−0.10	−0.08	0.22	0.17	<0.001
day_3_ – day_−3_	+0.03	+0.00	−0.09	−0.10	0.12	0.10	0.109
day_4_ – day_−3_	−0.08	−0.13	−0.14	−0.11	0.06	−0.02	0.718
day_5_ – day_−3_	−0.08	−0.08	−0.12	−0.12	0.03	0.04	0.554

Volume and position were determined from CBCT scans and expressed relative to the planning CT. Baseline volume and position were taken three days prior to start of hydration in the second chemotherapy cycle (week 4). Hydration started around treatment day 21, this day was renamed day 0. Significance (Mann–Whitney *U*-test) was tested against a control group receiving radiotherapy only. No significant results were found for the distance change between parotid glands in dorso-ventral and cranio-caudal direction

*PG(s)* parotid gland(s), *CRT* chemoradiotherapy group, *RT* radiotherapy alone group

At the day of the 35th fraction, the mean PG volume loss within the CRT group was 25 % ± 12 % (1SD) and within the RT alone group 24 % ± 10 %. The PGs shifted in medial direction; at the day of the 35th fraction, the distance between the PGs decreased with an average of 0.44 cm ± 0.26 cm within the CRT group and with an average of 0.51 cm ± 0.27 cm within the RT alone group. There was no significant difference between the two groups regarding these overall trends.

## Discussion

PG volumes temporarily increase during hydration in patients treated for H&N cancer with a high dose cisplatin regimen in combination with RT. Moreover, the medial shift is reversed just after the start of hydration. When hydration was stopped, no significant differences between the CRT and RT alone group, regarding both volume and position of the PGs, were measured anymore. Although the effects of hydration are temporary, in the context of adaptive RT, caution should be taken in timing a repeat CT scan. A repeat CT scan made at the time of hydration will not be representative for the remaining fractions with respect to the PGs.

Overall during treatment, the PG volume decreased and the PGs shifted in medial direction in both groups. Previous studies [1–9] described a similar result. Han et al. [5] performed a study including 5 patients who were treated for H&N cancer with helical tomotherapy with daily MVCT scans. The study described an average decrease of the PG volume of 1.1 % per treatment day. According to Bhide et al. [1] the average decrease in PG volume was 1.1 % per day in the first four weeks of treatment (31 % by week 4). For this study, 20 patients underwent a repeat CT scan in week 2, 3, 4 and 5 during RT. In the study of Lee et al. [7], 10 patients were included who were treated with helical tomotherapy with daily MVCT scans. The study showed a median decrease in volume of 0.4 to 1.3 % per day. In the study of Bhide et al. [1] it appeared that the largest decrease in volume takes place in the second week of the treatment. At the end of treatment, mean PG volumes can decrease with more than 50 %, i.e., Han et al. [5] found a decrease of 59.8 % ± 17.4 % of the original volume. The PG volume decrease with the patients of the current study is smaller.

Vasquez et al. [8] showed that the lateral regions of the glands move during RT while the medial regions are relatively fixed. This would imply that a change of the COM is more the result of volume change than an indicator of positional change. Still, a movement of lateral areas to a more medial position because of shrinkage could increase the mean dose to the organ. Han et al. [5] correlated an average decrease of the PG volume of 0.21 cm^3^ per treatment day with an average increase of the dose to the PGs of 1.7 cGy per treatment day. Lee et al. [6] correlated the COM change of the PGs with the percent difference between plan and cumulative mean dose. With the CRT group in the present study, decrease of the PG volume and medial movement is temporarily interrupted during hydration. Based on the study of Han et al. [5] and Lee et al. [6], hypothetically the fraction dose received by the PGs is temporarily reduced, with a limited effect on the total dose. However, Bhide et al. [1] demonstrated only a significant increase in mean dose to the ipsilateral gland at week 4. When the mean dose to the PGs were analyzed together it did not show a significant increase in mean dose.

Our study has a number of limitations. First, the fractionation schemes of the two groups differs. However, at the end of week three, three days before the start of prehydration of the second chemotherapy cycle, no significant differences between the CRT and RT alone group, regarding both relative volume and position of the PGs, were measured.

Second, it was only possible to verify the volume and position of the PGs during the second chemotherapy cycle (week four). Although comparable trends were seen during hydration of the first and third chemotherapy cycle, respectively first and seventh week of treatment, there wasn’t sufficient data for the applied method to confirm these findings. However, the timing of adaptive RT will predominantly be mid-treatment, making our findings relevant to clinical practice.

Third, the position of the PGs was specified with respect to each other. Shifts in dorso-ventral and cranio-caudal directions could be better specified if the position of the PGs is measured in comparison with an immobile structure, for example a bone structure. However, the applied method is adequate to determine changes in medial-lateral direction, which is the most relevant direction of positional change for the PGs.

Fourth, the planned dose on the ipsi- and contralateral PG within both groups showed large variations (Table 1). We did not take this into account in this study based on the results of Bhide et al. [1]. He distinguished no significant difference between the volume decrease of the ipsi- and contralateral PG. To test this with our data, we compared the relative volume of the ipsi- to the contralateral PG at the end of week three: no significant difference was found. This confirms the observation of Bhide et al. [1]. In addition, both the ipsi- and contralateral PG are subjected to the same amount of hydration. This justifies combining the ipsi- and contralateral PG volume for the purpose of the present study.

As a final limitation, we used DIR to propagate the delineations from the pCT to the CBCT scans. With DIR, uncertainties in accuracy are present. However, an independent validation of the DIR algorithm that was used in this study, by Mencarelli et al. [15] with gold markers around the tumor showed submillimeter accuracy. Whereas, the precision was around 3 mm. Since the precision is a random effect, this will not bias our results. Furthermore, according to Lee et al. [7], geometrical uncertainties of automatically generated contours are comparable to those of manual contours. They concluded that the deformable registration process provides a tool for automatic evaluation of anatomical PG changes occurring during treatment.

In this study, the influence of intravenous hydration, consisting of 9 l in three days in combination with a 3 weekly cisplatin based CRT treatment, was verified. Different amounts of fluid during hydration or a different CRT scheme could have a different influence on volume and position of PGs. Moreover, there are several other factors that could be considered of having a possible influence on the geometry of the PGs. For example: tumor volume, tumor site, cytostatics, feeding status, BMI and gender [1, 3–5, 16, 17]. These factors are not investigated in this study. Possible influence of tube feeding on the geometry of the PGs also is not investigated in this study.

## Conclusions

This study confirms overall PG shrinkage during (C)RT and an overall decrease of the mean medial distance between the PGs. Hydration during high dose cisplatin based CRT treatment of patients with H&N cancer, results in an increase of the PG volume in combination with a lateral movement of the COM. These effects of hydration are small and temporary. Unless, in the context of adaptive RT, a repeat CT scan is timed during a chemotherapy cycle, these effects are of minor clinical relevance. However, since the effect of hydration on PG geometry has not been investigated before, these results are relevant to share with the research community.

## Abbreviations

CBCT: Cone Beam CT
COM: Center-of-mass
CRT: Chemoradiotherapy
DIR: Deformable image registration
H&N: Head and neck
OARs: Organs-at-risk
pCT: Planning CT
PG: Parotid gland
PGs: Parotid glands
RT: Radiotherapy
